# Reversine inhibits Colon Carcinoma Cell Migration by Targeting JNK1

**DOI:** 10.1038/s41598-018-30251-w

**Published:** 2018-08-07

**Authors:** Mohamed Jemaà, Yasmin Abassi, Chamseddine Kifagi, Myriam Fezai, Renée Daams, Florian Lang, Ramin Massoumi

**Affiliations:** 1 Department of Laboratory Medicine, Translational Cancer Research, Lund University, Lund, 22381 Sweden; 2 Department of Physiology I, Tübingen University, Gmelinstr. 5, D-72076 Tübingen, Germany; 3 Division of Immunology and Vaccinology, Technical University of Denmark, Copenhagen, Denmark; 4 Department of Molecular Medicine II, Medical Faculty, Heinrich Heine University, Duesseldorf, Germany

## Abstract

Colorectal cancer is one of the most commonly diagnosed cancers and the third most common cause of cancer-related death. Metastasis is the leading reason for the resultant mortality of these patients. Accordingly, development and characterization of novel anti-cancer drugs limiting colorectal tumor cell dissemination and metastasis are needed. In this study, we found that the small molecule Reversine reduces the migration potential of human colon carcinoma cells *in vitro*. A coupled kinase assay with bio-informatics approach identified the c-Jun N-terminal kinase (JNK) cascade as the main pathway inhibited by Reversine. Knockdown experiments and pharmacological inhibition identified JNK1 but not JNK2, as a downstream effector target in cancer cell migration. Xenograft experiments confirm the effect of JNK inhibition in the metastatic potential of colon cancer cells. These results highlight the impact of individual JNK isoforms in cancer cell metastasis and propose Reversine as a novel anti-cancer molecule for treatment of colon cancer patients.

## Introduction

Colorectal cancer (CRC), a tumor on the inner lining of the rectum or colon is one of the most common cancers and a major cause of cancer-related death worldwide^1,2^. Despite substantial improvement in CRC diagnosis and therapy, the survival of CRC patients remains poor due to cancer cell metastasis^3^. Thus, development and characterization of inhibitors counteracting CRC metastasis are needed.

The JNK pathway is one of the six distinct groups of MAPK (Mitogen-activated protein kinase)^4^. Three genes encode the family; *Jnk1*, *Jnk*2 and *Jnk3*. *Jnk 1* and *2* are ubiquitously expressed, whereas *Jnk3* expression is restricted to the brain, heart and testis^5^. JNK is activated in response to various extracellular stimuli, including tumor necrosis factor (TNF), epidermal growth factor (EGF), platelet-derived growth factor (PDGF), transforming growth factor β (TGF-β) and lysophosphatidic acid, as well as diverse environmental stresses^6^. The JNK pathway plays a key role in cell differentiation, inflammation and apoptosis^4^. Moreover, JNK is implicated in cell migration of several cell types^6^. The role of JNK signaling in CRC has been well documented recently with increasing evidence in support of up-regulated JNK activation in intestinal tumors. Indeed, activation of JNK in the intestine promotes cell proliferation^7^. Moreover, two well-known tumor suppressor genes; FBXW7 (F-box/WD repeat-containing protein 7) and PDCD4 (programmed cell death 4) that can inhibit the activity of JNK, were shown to be inactivated in CRC^8–10^. Along those lines, pharmacologic inhibition of JNK reduced the growth of several adenocarcinoma cell lines^7,11^. Furthermore, JNK1/c-jun pathway is involved in multidrug resistance of colon cancer cells^12^.

In a previous work, we showed that two mitotic kinase inhibitors namely SP600125 and Reversine reduced the migration of soft tissue sarcoma cell lines^13^. SP600125 (anthra[1,9-cd]pyrazol-6-(2H)-one), a reversible ATP-competitive inhibitor of MAPK-JNK, was identified as direct inhibitor of JNK activity in a high throughput screening of a private chemical library held by Celgene^14^. SP600125 targets specifically JNK1, JNK2 and JNK3 with an IC_50_ values of 40 nM for JNK1 and JNK2, as well as 90 nM for JNK3^14^. SP600125 further inhibits the mitotic serine/threonine kinases Aurora kinase A and B^15^ and Monopolar spindle 1 kinase (Mps1)^16,17^. SP600125 is widely used to disrupt signaling underlying diverse biological processes including inflammation, neurodegeneration, metabolic disease and cancer^18^.

Reversine (2-(4-morpholinoanilino)-6-cyclohexylaminopurine) is a small molecule synthesized at Scripps Research Institute California in 2003 and was first used as a dedifferentiation agent^19^. Reversine reverses differentiation of lineage-committed cells to mesenchymal stem cells (MSCs) allowing the cells to undergo differentiation into other lineages^20–22^. Later, the role of Reversine in anti-tumor activities, including mitotic catastrophe, cell-cycle arrest, polyploidy and autophagy was discovered in several cancer cell lines^23–27^. Recently, Reversine was reported as an inhibitor of eryptosis, the suicide of erythrocytes^28^. Structurally, Reversine is an ATP analogue and inhibits cellular enzymatic activities^29^ of Monopolar spindle 1 (Mps1) kinase^23,30,31^, Aurora kinase A and B^30–32^ and Akt/mTOR^33,34^.

In this study, we identified Reversine as a potent inhibitor of colon cancer cells migration and metastasis. The substance is effective by interference with JNK1-signaling.

## Results

### Reversine and SP600125 inhibit colon carcinoma cell migration

In a previous study, we developed a screening assay using the two dimensional Oris^TM^ cell migration to target invasive sarcoma cell lines by treating with several mitosis and cytoskeleton inhibitors at 3 different doses (0.1, 1 and 10 μM) for 24 hours^13^. The aim of this test was to discover potent anti-migratory agent/s (≥50% as a cut off) without any major cell toxicity (≤30% of toxicity as limit) (Fig. S1). By calculating both the ratio of the field area and cells count of the migration zone it was found that Reversine and SP600125 at 10 μM prevent the migration of sarcoma cells^13^. Thus, we decided to investigate the effect of these molecules on the migration of the human colon carcinoma cell line RKO which are considered as one of the most invasive colorectal carcinoma cell lines^35^.

**Figure 1:**
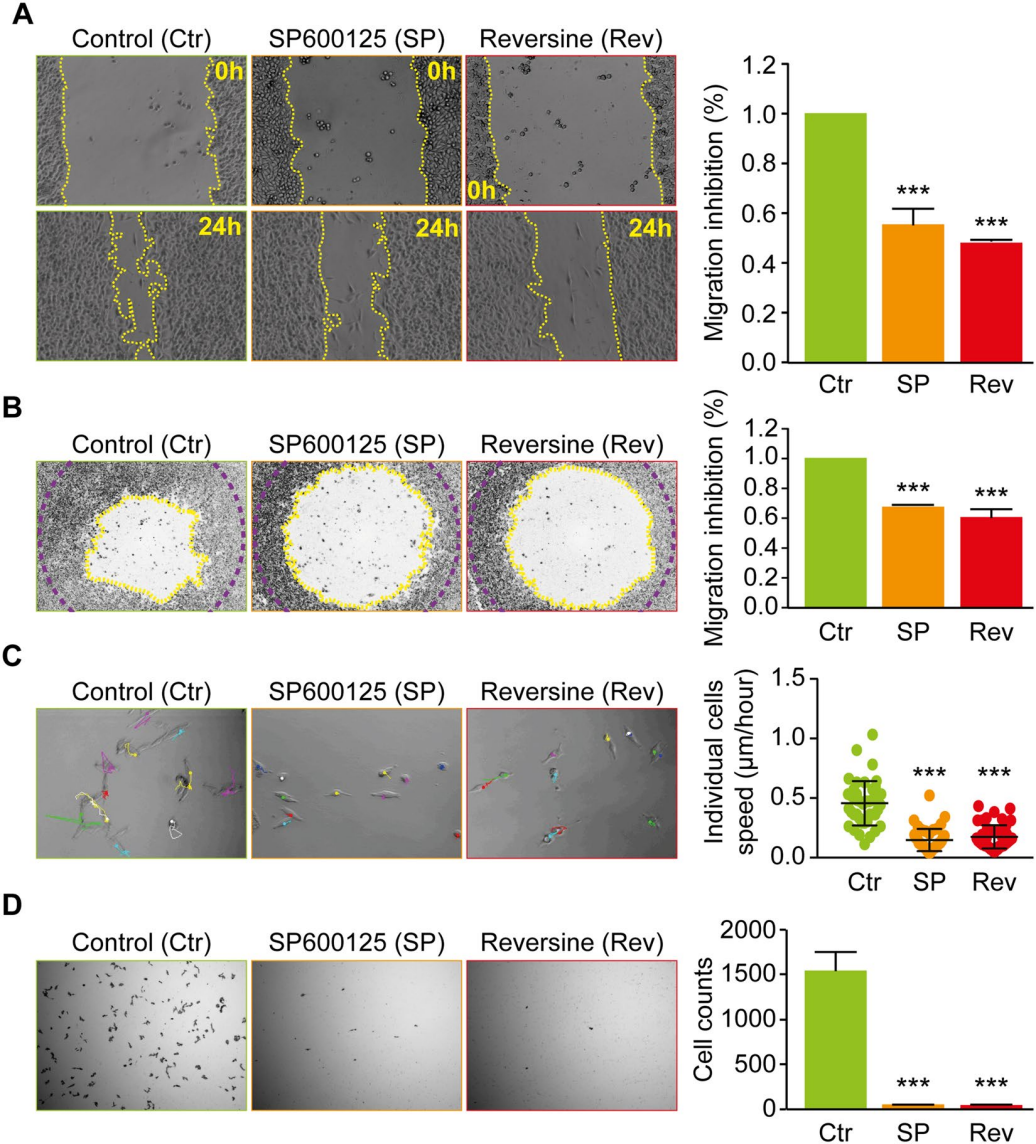
Reversine and SP600125 inhibit human colon carcinoma RKO cells migration. **(A)** Wound-Healing assay. The human colon carcinoma RKO cell lines were plated in a 6-well plate and kept to monolayer confluence and then a wound-healing assay was performed. Representative photomicrographs are shown. The yellow broken lines delimit the cell-free area. Quantitative data of anti-migration % comparatively to control are presented in the right of the panel. (**B**) Two-dimensional migration assay using the Oris™ cell assay. Cells were allowed to migrate for 24 h after the removal of cell seeding stoppers and treatment, fixed and stained with phalloidin and DAPI to evaluate their motile potential. Representative photomicrographs are shown. The yellow broken lines delimit the cell-free area after 24 h of migration while the purple broken lines delimit the cell-free area at time 0 h. Quantitative data are presented in the right of the panel. (**C**) Individual cell tracking assay. Cells were grown in non-confluent conditions and imaged for 24 hours, using time-lapse microscopy, to evaluate respective migration potential of individual cells. Representative photomicrographs are shown with the trajectories reconstituted using Image J software of some cells with or without treatment. Quantitative data of cells speed are presented in the right of the panel. (**D**) Boyden chamber assay. Cells were collected, washed and suspended in free serum medium. Cells were then added to the upper compartments of the Boyden chamber in the presence or absence of Reversine or SP600125. Representative photomicrographs of cells that had migrated 24 h later to the lower side of the filter are shown. Quantitative data are presented in the right of the panel. Data are reported in SEM; n = 3. ***(p < 0.001) indicates significant difference from the absence of Reversine or SP600125 treatment (ANOVA).

In a first experimental approach, we performed a wound-healing test and found that the decrease of cell free area was significantly delayed in the presence of SP600125 (55 +/− 0.7%) or Reversine (48 +/− 0.1%) (Fig. 1A). The two dimensional Oris^TM^ cell migration assay confirmed the anti-migratory effect of Reversine and SP600125 compared to solvent treated RKO cells (Fig. 1B), Reversine inhibited migration by 40 +/− 0.1% and SP600125 by 37 +/− 0.1%. In addition, SP600125 and Reversine reduced individual cell migration, tracked by time laps microscopy (Fig. 1C). Moreover, using Boyden chamber assay, cell invasion was completely abolished in cells treated with Reversine and SP600125 for 24 h (Fig. 1D)

**Figure 2:**
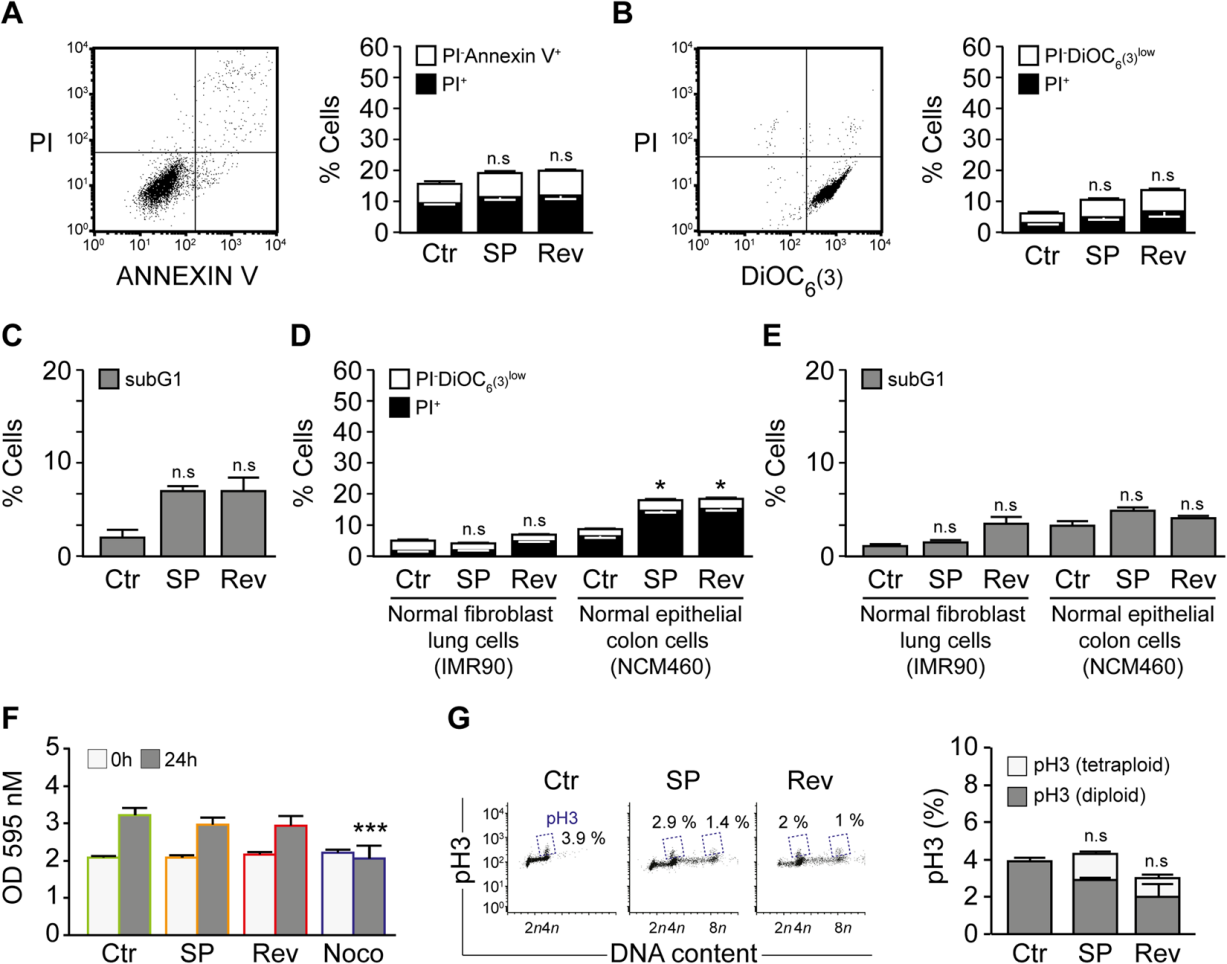
Inhibition of migration by Reversine and SP600125 is not due to treatment toxicity.(**A–C**) The toxicity of Reversine and SP600125 at 24 h was evaluated by flow cytometry upon staining with the cell death–associated parameters dyes. (**A**) Human colorectal carcinoma RKO cells were left untreated or treated for 24 h with 10 μM SP600125 or Reversine and then co-stained with the vital dye propidium iodide (PI) and the FITC-conjugated Annexin V for the detection of phosphatidylserine exposure. Representative dot plot (SP600125 treatment) and quantitative data are reported. White columns depict the percentage of dying cells (PI^−^Annexin V^+^) while black columns illustrate dead cells (PI^+^). (**B**) Cells were co-stained with the vital dye propidium iodide (PI) and the mitochondrial membrane potential (Δψm)-sensing dye DiOC_6_(3). Representative plot of SP600125 treated cells is shown and quantitative data are represented. White and black columns depict the percentage of dying (PI^−^DiOC_6_(3) ^low^) and dead (PI^+^) cells, respectively. (**C**) RKO cells left untreated or exposed for 24 h with SP60015 or Reversine were fixed with ethanol and labeled with the DNA dye PI, for the quantification of the subG1 apoptotic population. Quantitative data are reported. (**D–E**) The toxicity of Reversine and SP600125 on non-cancer cells line. (**D**) Non transformed fibroblast IMR90 and normal human colon mucosal epithelial cell line NCM460 were left untreated or treated for 24 h with 10 μM SP600125 or Reversine and then co-stained with the vital dye propidium iodide (PI) and the mitochondrial membrane potential (Δψm)-sensing dye DiOC_6_(3). Quantitative data are represented. White and black columns depict the percentage of dying (PI^−^DiOC_6_(3) ^low^) and dead (PI^+^) cells, respectively. (**E**) IMR90 and NCM460 cells left untreated or exposed for 24 h with SP60015 or Reversine were fixed with ethanol and labeled with the DNA dye PI, for the quantification of the subG1 apoptotic population. Quantitative data are reported. (**F–G**) Reversine and SP600125 do not affect cells proliferation and induces polyploidization. (**F**) Cells were seeded in 96 well plates and cultured for 24 h with or without treatment. Nocodazole at 200 ng/ml was used as positive control. Cell proliferation was assessed using crystal violet assay. Quantitative data are shown. Grey bar depict the optic density at 0 h while dark grey bar represent the optic density at 24 h, respectively. (**G**) Cells left untreated or exposed for 24 h with SP60015 or Reversine were fixed with ethanol and stained with an antibody specific for phosphorylated histone 3 (pH3), which is a mitosis-specific marker. Representative scatter plots and quantitative results of pH3 are shown. Numbers indicate the percentage of cells found in each quadrant. Data are reported in SEM; n.s indicates non-statistical difference from the control (ANOVA). *(p < 0.05) indicates statistical difference from the control. ***(p < 0.001) indicates statistical difference from the absence of Nocodazole treatment (ANOVA).

To exclude toxic effects of Reversine and SP600125, the RKO cells were treated with Reversine and SP600125 for 24 hours and subsequently used for Flow Cytometry (FACS) analysis to assess cell death associated parameters. The cells were co-stained with the propidium iodide to check plasma membrane integrity, and Annexin V-FITC to measure cell membrane scrambling and phosphatidylserine translocation, which are used as a hallmark of apoptosis. Cell membrane scrambling and phosphatidylserine outer membrane exposure was similar in Reversine and SP600125 treated cells and DMSO-treated cells (Fig. 2A**)**. Further, we used DiOC_6_(3), to measure the mitochondrial transmembrane potential (Δψm). Mitochondrial potential and cell membrane permeability were not significantly different between Reversine or SP600125 treated cells and DMSO treated cells (Fig. 2B). Moreover, cell cycle FACS analysis showed that subG1 fraction relative to DNA degradation was not significantly different between SP600125 or Reversine treated cells and DMSO-treated cells (Fig. 2C). Together, these results suggest that 24 h treatment of colon cancer cells with Reversine and SP600125 does not affect cell death parameters. In parallel, we decided to investigate the cytotoxicity effect of Reversine and SP600125 treatment in non-cancerous cells including non-transformed fibroblast IMR90 and normal human colon mucosal epithelial cell NCM460 using the propidium iodide and DiOC_6_ staining. It was found that mitochondrial potential and cell membrane permeability were not different between treated and control cells (Fig. 2D). The evaluation of the subG1 fraction further confirmed this finding (Fig. 2E). Together, these results suggest that 24 h treatment of non-cancerous cells with Reversine and SP600125 does not affect cell death parameters.

To exclude any anti-proliferative effect of Reversine or SP600125 that may overlap with the anti-migration effect, several assays were performed. Crystal violet cell proliferation assay showed that Reversine and SP600125 has no influence in cell proliferation after 24 h of treatment (Fig. 2F and Fig. S2). *A contrario*, treatment of cells with 200 ng/ml Nocodazole, which we used here as a potent anti-mitotic drug, showed a complete proliferation arrest (Fig. 2F). Next, we analyzed and compared the status of cell cycle in Reversine and SP600125 treated cells compared to DMSO. Treatment of cells Reversine and SP600125 continued to cycle and undergo polyploidy as well as exhibited histone H3 phosphorylation, which is a marker of ongoing cell mitosis (Fig. 2G). Altogether, this data together with our previous findings demonstrate that SP600125 and Reversine do not influence cancer cells proliferation at 24 h but instead induces cells polyploidization^17,23^.

### Reversine and SP600125 target the JNK pathway

**Figure 3:**
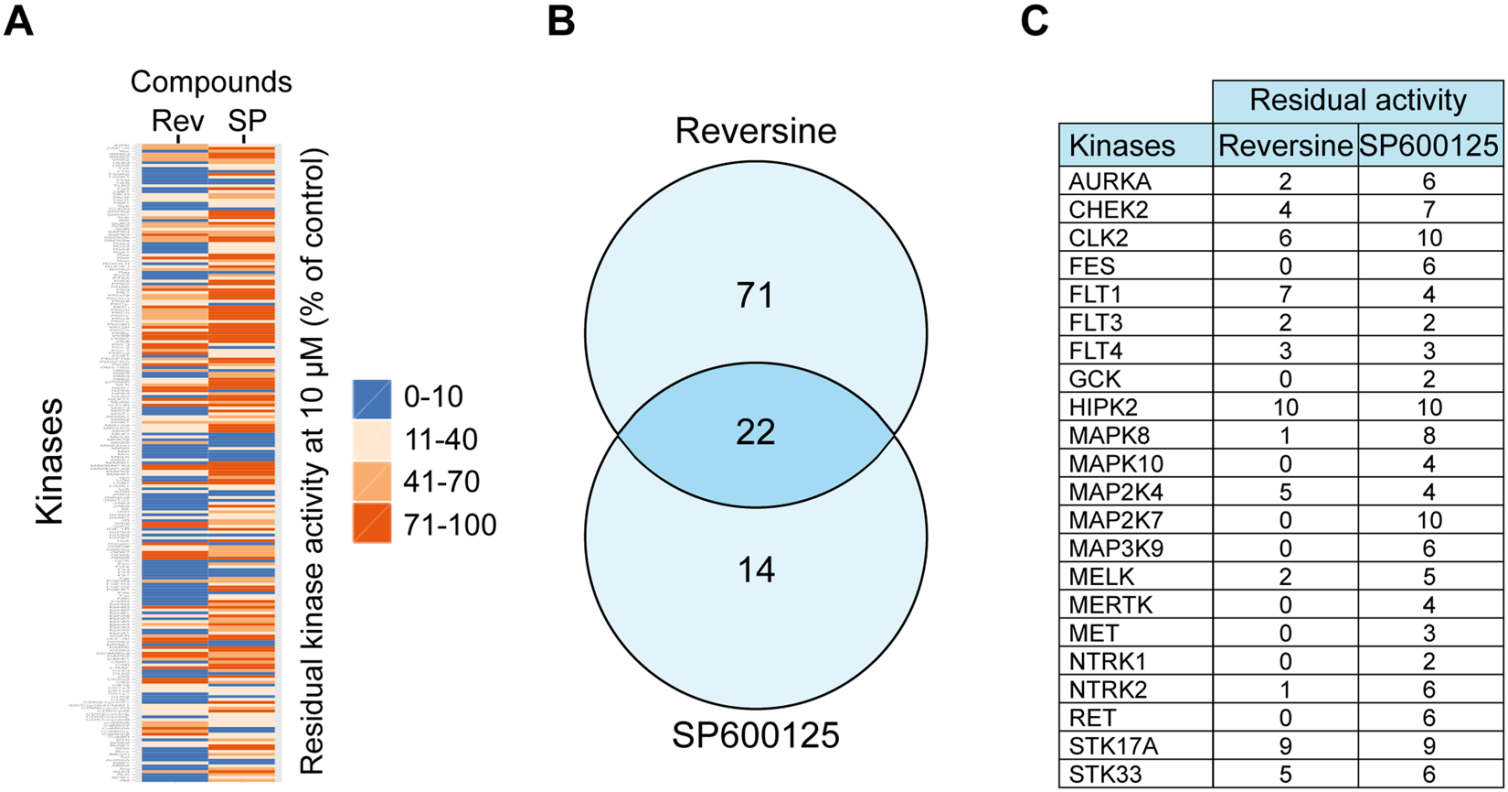
Identification of Reversine and SP600125 common targets. (**A–C**). A kinase profiler screen of cells treated with 10 μM Reversine (Rev) or 10 μM SP600125 (SP) was analyzed. The heat map in panel (**A**) shows the residual kinase activity (% of control). The scale ranges from 0% (blue) to 100% (red). The Venn diagram in panel (**B**) displays the proteins that were inhibited more than 90% by either 10 μM of Reversine or SP600125. The 22 common target proteins are listed in panel (**C**).

Reversine and SP600125 are reported as broad-spectrum inhibitors of serine/threonine kinases^17,23^. To identify the common target kinases of these small molecules, we decided to perform a kinase profiler screening^36^ (Fig. 3). Treatment of RKO cells with 10 µM of Reversine or SP600125 inhibited several kinases with varying efficiency. We selected kinases that were inhibited more than 90%. As a result, in a panel of 222 kinases, SP600125 and Reversine inhibited 36 and 93 kinases, respectively. However, 22 kinases were inhibited by both drugs (Fig. 3B–C). Focusing on these 22 kinases, and to select the pathway involved in the anti-migration effect of SP600125 and Reversine, we choose a bio-informatics approach. Genes relative to the kinases list were used in enrichment analysis to identify over-represented gene ontology (GO) categories (Fig. S3).

**Figure 4:**
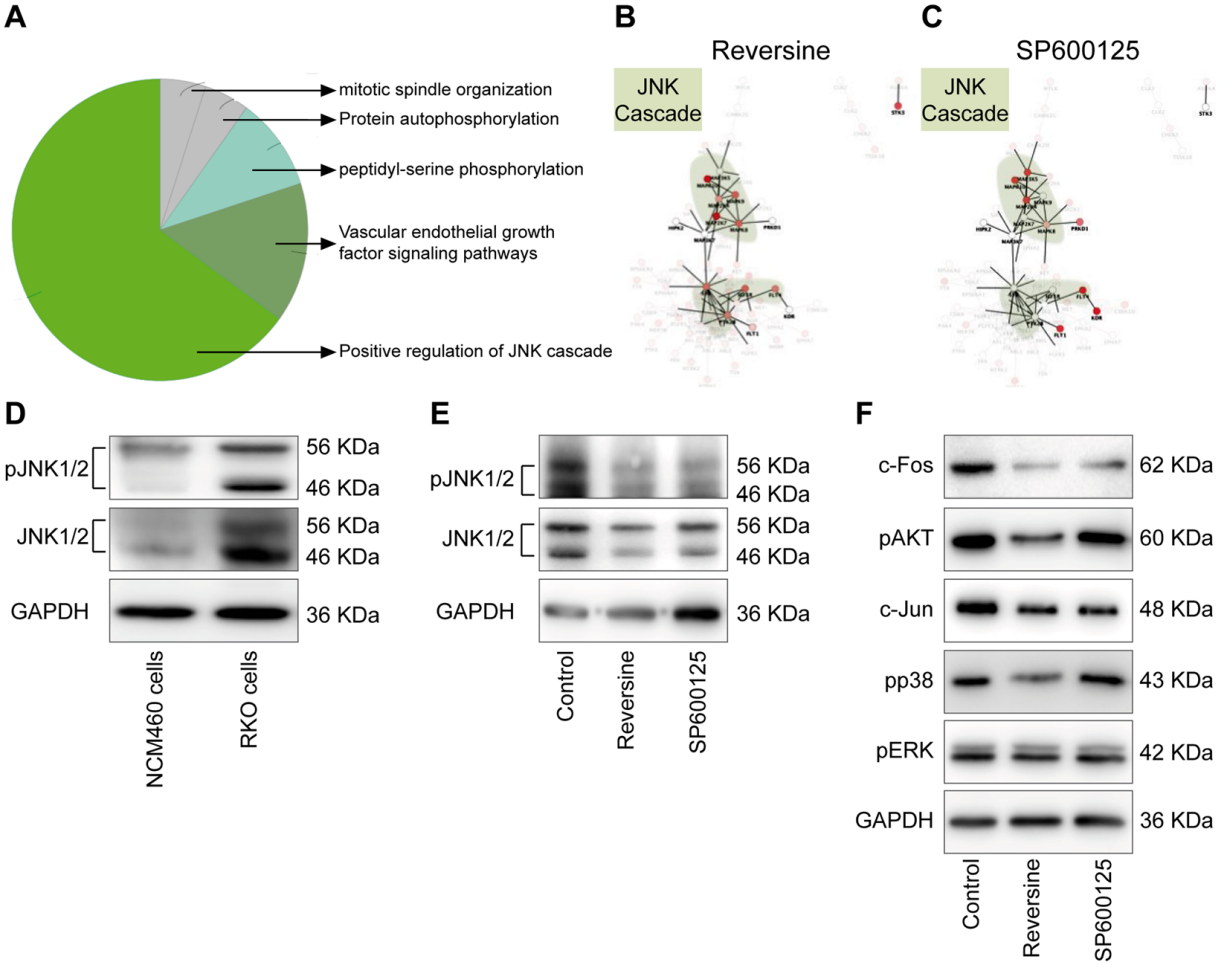
The JNK cascade is the principal pathway inhibited by Reversine and SP600125. (**A–C**) Gene Ontology analysis of the common target genes (22) of Reversine and SP600125 was done by the Cytoscape associated-plugin ‘ClueGO’. ‘Positive regulation of JNK cascade’ was the most associated term with both Reversine and SP600125 (**A**). Network view of target genes of Reversine and/or SP600125 was performed by Cytoscape and GeneMANIA associated-plugin. Red colors represent the inhibited proteins through the Kinase panel test and JNK related nodes were highlighted in green zones after Reversine (**B**) and SP600125 (**C**) treatment. (**D**) Overexpression and activation of JNK was investigated in RKO colon cancer cell line comparatively to the normal human colon mucosal epithelial cell line NCM460. (**E**) Inactivation of JNK by SP600125 or Reversine in RKO cells was confirmed by Western blot. (**F**) Downstream targets of JNK pathway after treatment with Reversine or SP600125 were checked by western blot.

Regulation of the JNK cascade exhibited the highest enrichment score (*Term* p-value after correction with Bonferroni = 1,42 × 10^−6^) for the common SP600125 and Reversine-inhibited kinase (Fig. 4A). The other top gene-sets were mitotic spindle organization, protein auto-phosphorylation, peptidyl-serine phosphorylation, and vascular endothelial growth factor (VEGF) signaling pathways (Fig. 4A–C). To confirm the role JNK in the migration process, we decided to investigate the expression and activation of JNK in the RKO cells compared to the normal human colon mucosal epithelial cell NCM460. It was found that JNK is highly expressed and activated in the human colon cancer cells compared to the non-cancerous cells (Fig. 4D). Moreover, by checking the level of total JNK and phospho JNK in control *vs*. treated cells with Reversine or SP600125, we found that Reversine affect slightly the expression of JNK protein, particularly the levels of JNK1 (The lower band in the western corresponding to 46 KDa, Fig. 4E). Furthermore, Reversine or SP600125 treatment completely abolishes the phosphorylation of JNK1/2. (Fig. 4E). Next, we investigated the downstream targets of JNK pathway upon Reversine treatment. Reversine decreased the expression of c-Fos and c-Jun, a direct target of JNK-mediated pathway (Fig. 4F)^37^. Interestingly, Reversine also inhibited activation of p38 but not ERK. We also evaluated the level of Akt activation (another player of cell migration)^38^ and confirmed the Reversine selectivity, *a contrario* of SP600125, for Akt^39^ (Fig. 4F).

### Inhibition of JNK1 but not JNK2 reduces the migratory potential of colon carcinoma cells

**Figure 5:**
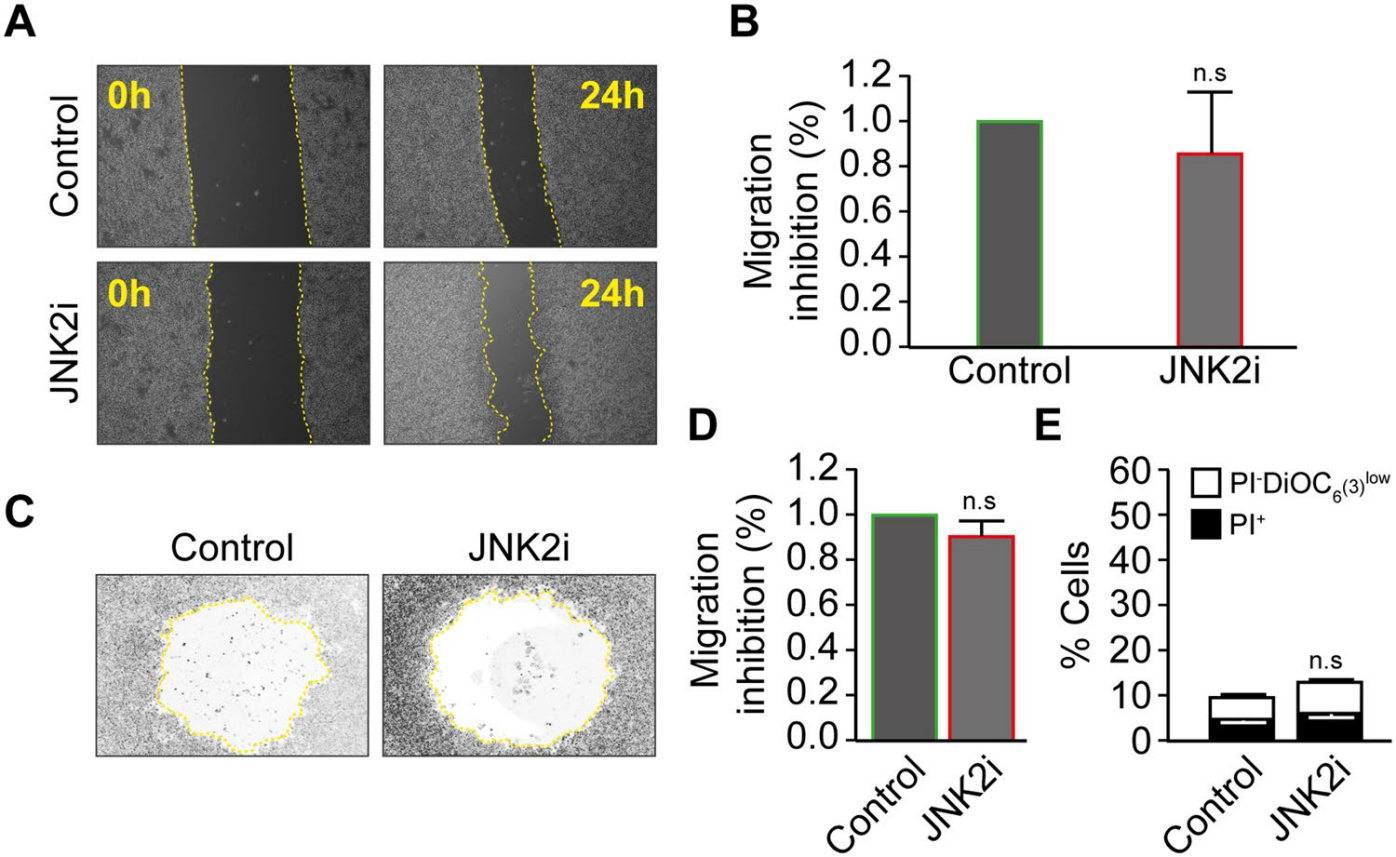
Specific inhibition of JNK2 does not affect the migratory potential of the human colon carcinoma RKO cells. (**A**,**B**) Wound-Healing assay. The human colon carcinoma RKO cell was plated in a 6-well plate and kept to monolayer confluence and then a wound-healing assay was performed. Representative photomicrographs are shown in panel (**A**), the yellow broken lines delimit the cell-free area. Quantitative data of anti-migration % comparatively to control are presented in panel (**B**). (**C**,**D**) Two-dimensional migration assay using the Oris™ cell assay. Cells were allowed to migrate for 24 h after the removal of cell seeding stoppers and treatment, fixed and stained with phalloidin and DAPI to evaluate their motile potential. Representative photomicrographs are shown in panel **(C)**, the yellow broken lines delimit the cell-free area after 24 h of migration. Quantitative data are presented in panel **(D)**. (**E**) The toxicity of JNK2 inhibitor was evaluated by flow cytometry upon staining with the vital dye propidium iodure (PI) and the mitochondrial membrane potential (Δψm)-sensing dye DiOC6(3). Data are reported in SEM; n = 3. n.s indicates non-statistical difference from the control (ANOVA).

**Figure 6:**
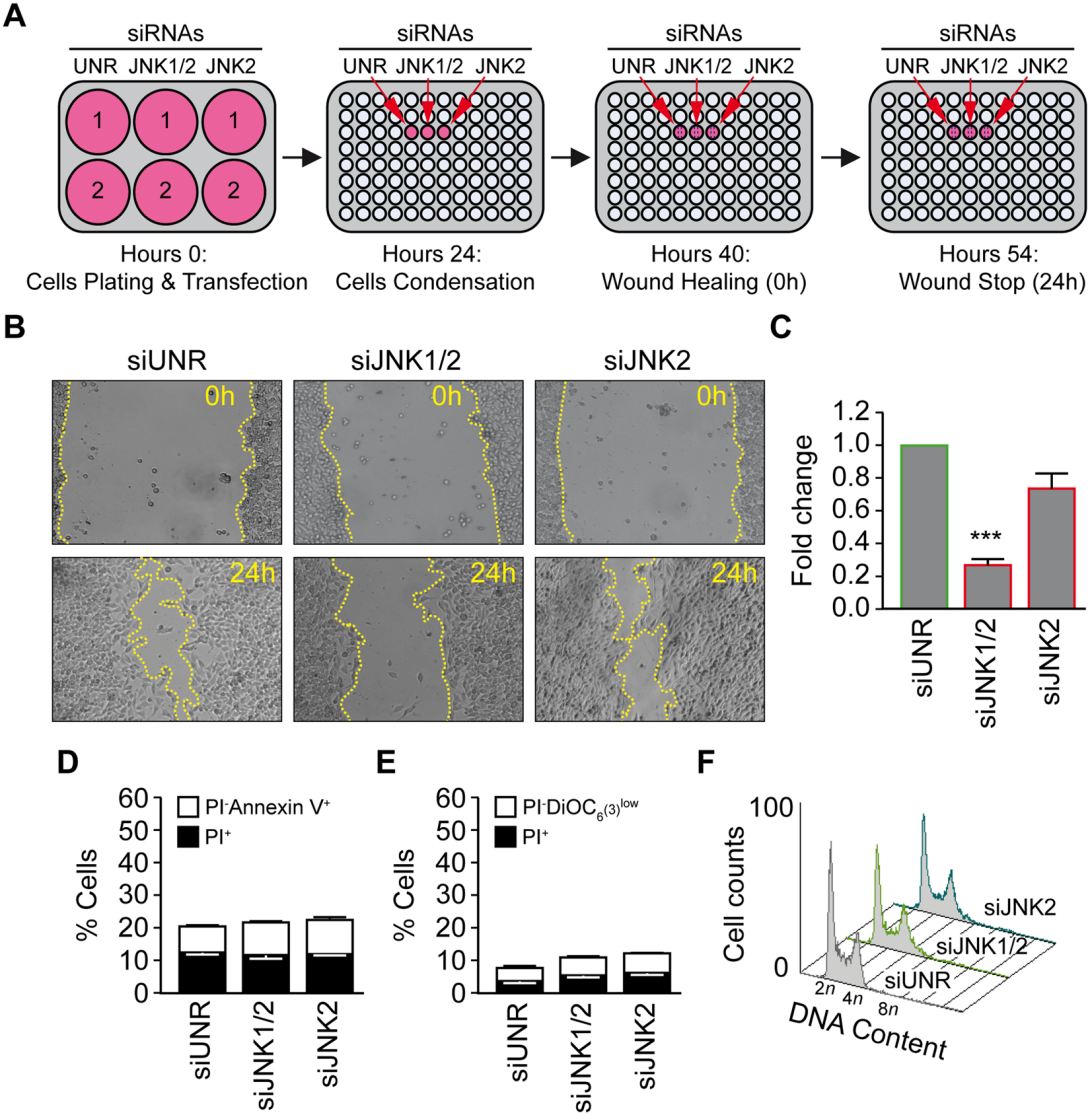
Inhibition of JNK1 and not JNK2 reduces the migratory potential of the human colon carcinoma RKO cells. (**A–C**) Cells were seeded into 6-well plate and were transfected with an unrelated small interfering (si) RNA (siUNR) or specific siRNAs directed against JNK1/2 or JNK2 (siJNK1/2 and siJNK2) (two well per condition). Upon 24 h, cells were harvested and seeded in a 96-well plate to ensure a monolayer confluence. After overnight culture a wound-healing test was performed and migration was evaluated after 24 h. Representative photomicrographs are shown in panel (**B**), the yellow broken lines delimit the cell-free area. Quantitative data of anti-migration % comparatively to control are presented in panel (**C**). (**D-E**) The toxicity of siRNAs JNK1/2 or JNK2 was evaluated by flow cytometry upon staining with the cell death–associated parameter dyes. RKO cells were transfected with siUNR, siJNK1/2 or siJNK2 for 48 h and then co-stained with the vital dye propidium iodide (PI) and the FITC-conjugated Annexin V for the detection of phosphatidylserine exposure (**D**) or co-stained with the vital dye propidium iodide (PI) and the mitochondrial membrane potential (Δψm)-sensing dye DiOC_6_(3) (**F**). Cells were transfected with siUNR, siJNK1/2 or siJNK2 for 48 h and then fixed with ethanol and labeled with the DNA dye PI, for the quantification of cell cycle. Representative histograms are shown. Data are reported in SEM; n = 3. ***(p < 0.001) indicates significant difference from the siUNR transfection (ANOVA).

Three kinases, JNK1 (MAPK8), JNK2 (MAPK9), and JNK3 (MAPK10), constitute the c-Jun N-terminal kinase family (JNK)^40^. In our study, we excluded JNK3 since it is mainly expressed in neurons, heart and testis. To distinguish between JNK1 and JNK2-mediated cell migration, we treated cells with a specific inhibitor of JNK2 (JNK inhibitor IX, Santa Cruz Biotechnology, Dallas, USA)^41,42^ and performed a wound-healing (Fig. 5A,B) as well as a two dimensional Oris^TM^ cell migration assay (Fig. 5C,D). Wound recovering and migration zone invasion were similar after 24h-treatment with the JNK2 inhibitor and control treated cells. To confirm this data, we treated the cells with small interfering RNAs specific for JNK1 or JNK2. As depicted in Fig. 6A, RKO cells were transfected with unrelated siRNA (siUNR), siJNK1/2, or siJNK2 in a 6-well plate for 24 h and condensed further into a 96-well plate. The efficacy of siRNA JNK1/2 and JNK2 were studies using Western blot analysis (Fig. S4). After the cells formed a monolayer, a scratch assay was performed, and cells migration was monitored by microscopy. It was evident that the decline of cell free area was significantly delayed following knockdown of JNK1/2 compared to siRNA UNR (74 +/− 0.4% of inhibition) or siJNK2-treated cells (Fig. 6B,C). To exclude toxic effect of JNK1 and JNK2 knockdown, FACS analyses of the death related parameters were investigated. It was found that siRNA transfection of RKO cells with JNK1 or JNK2 did not induce any significant changes in the overall cell survival (Fig. 6D,E). Moreover, we analyzed the cell cycle of RKO after the knockdown of JNK1/2 and JNK2 and found that the siRNA transfection was not influencing cell cycle or cell proliferation (Fig. 6F).

### The administration of SP600125 *in vivo* affects the metastasis of human colon carcinoma RKO cells

**Figure 7:**
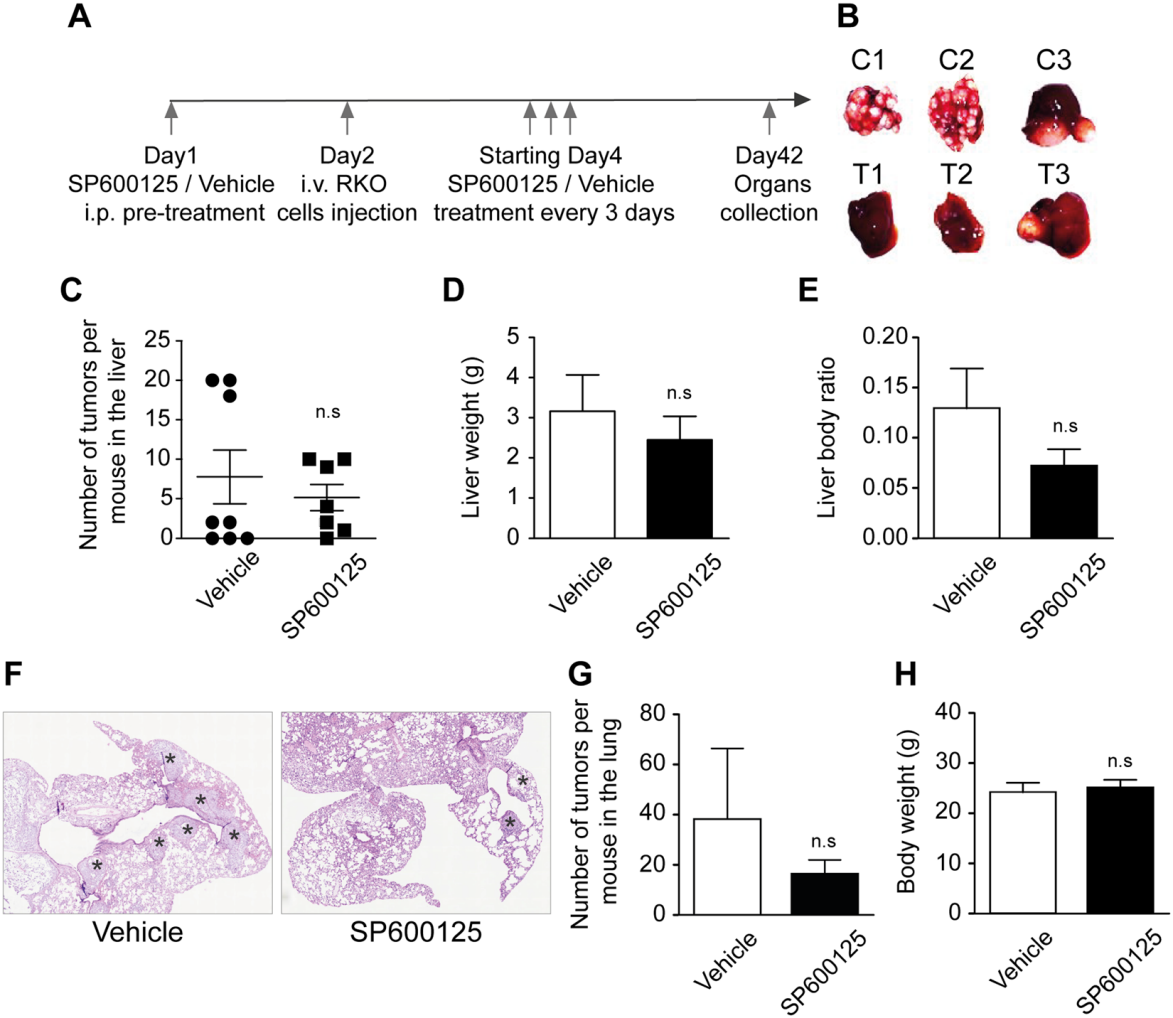
SP600125 reduces the metastasis of the human colon carcinoma RKO cells *in vivo*. (**A**) NSG mice were pre-treated i.p. (Intra peritoneal) with a control vehicle (n = 8) or 10 mg/kg SP600125 (n = 7). The day after, RKO cells were injected intravenously (i.v.). Treatment was performed every 3 days. Mice were sacrificed after 8 weeks and tumors were counted. (**B**) Representative pictures of liver in vehicle (C1, C2 and C3) and SP600125 treated mice (T1, T2, and T3) respectively. (**C–E**) The number of tumors per liver in the mice treated with the vehicle or SP600125 is depicted in (**C**) while liver weight and the ratio of the liver weight per mouse are showed in (**D**) and (**E**), respectively. (**F**) HE staining for quantification of tumor incidence in the lung of mice treated with the vehicle or SP600125. Stars indicate tumors. (**G**) Number of tumors per lung. (**H**) The weight of mice in control *vs* SP600125. n.s indicates non-statistical difference from the control (T-test).

To evaluate the therapeutic and anti-metastatic potential of JNK inhibition *in vivo*, animals were injected intravenously with 2 × 10^6^ RKO cells/mouse. The mice were treated intraperitoneally every 3 days with a control vehicle or 10 mg/kg SP600125 (Fig. 7A**)**. The SP600125-treated mice showed fewer tumor metastases in the liver (Fig. 7B,C), lung (Fig. 7F,G**)**, spleen, and kidney (Data non shown) compared with control-treated animals. Moreover, the average and ratio of liver weight compared to the body weight were higher in vehicle- compared to SP600125-treated mice (Fig. 7D,E). No differences could be observed in the average body weight comparing vehicle and SP600125-treated mice (Fig. 7H). To further confirm the implication of JNK inhibition *in vivo*, we extracted the proteins from tumors isolated from lung and liver for western blotting. Densitometic analysis demonstrated that SP600125 reduces phospho JNK level in treated lung and liver comparatively to control. (Fig. S5).

## Discussion

Metastasis is the leading cause of cancer mortality including colon cancer, which is one of the most invasive and metastable tumors^43^. Thus, it is very important and urgent to develop anti-cancer drugs to reduce tumor cells dissemination. Here, we report the identification of the small molecules Reversine and SP600125, which can interfere with JNK signaling, as inhibitors of migration and metastasis of colon carcinoma cells. In our study, we decided to use the primary colon cancer RKO cell line which harbors the microsatellite instability (MSI) phenotype and represents 15% of all CRCs. RKO cells harbor mutations in KRAS, BRAF, PIK3CA and PTEN and are considered as one of the most invasive colon cancer cell lines^44^.

We took advantage of a novel screen assay, using the two dimensional Oris^TM^ cell migration developed in a previous study^13^. The previous test revealed Reversine and SP600125 as potent anti-migratory drugs of invasive sarcoma cells. In the present study, we explored the effect of Reversine and SP600125 on the human colon carcinoma RKO cells. We exposed cells for 24 h to both small molecules and found that this treatment significantly delayed the migration and invasion of RKO cells using different assays, namely wound-healing, two dimensional Oris^TM^ assay, individual cell tracking assay, and Boyden chamber assay. The anti-migratory effect of these drugs was not due to toxicity or cell apoptosis. Moreover, the treatment did not affect cell proliferation at 24 h, which is in line with our previous findings^17,23^. In this work we could also show that 24 h treatment with Reversine or SP600125 is non-toxic to the non-cancerous cells including non-transformed fibroblast and human colon mucosal epithelial cells. The kinase profiler screening identified several kinases that were inhibited by both Reversine and SP600125. This result in combination with bio-informatics (GO: gene ontology) approaches identified the JNK family activation as the most significant pathway inhibited by Reversine and SP600125. To distinguish between JNK1 and JNK2 activation, RKO cells were treated with specific siRNAs targeting JNK1/2 or JNK2, as well as treatment with a specific JNK2 inhibitor. The results demonstrated that only JNK1 inhibition delays the motility of these cells. Western blot analysis showed that JNK is overexpressed and activated in RKO cancer cells compared to normal colon mucosal epithelial cells. We could also show that Reversine, similar to SP600125, reduces the activation and expression of JNK in treated cells. Additionally, we demonstrate that activation of JNK related signaling pathway proteins including c-Fos, c-Jun, and p38 but not ERK were significantly decreased with Reversine treatment. The specificity of Reversine but not SP600125 could be observed against the activation of AKT, which is in line with previous finding^39^.

Reversine is a specific inhibitor of the human mitotic kinase Mps1^30^, whereas SP600125 is a well-known inhibitor for JNK1/2^14^. Reversine inhibits Mps1 *in vitro* with an IC50 of 63 nM^45^, whereas SP600125 targets Mps1 with an IC50 of 1.95 µM and JNK with an average of 56 nM (JNK1, 2 and 3)^16^. Previous studies could show that Mps1 and JNK have a high degree of sequence similarity in their ATP-binding pocket^16,46^. Mutation of methionine M108 to glutamine in JNK1 renders it insensitive to SP600125 inhibition^47^. Moreover, the corresponding mutation in Mps1 (M602Q) also proved significantly less sensitive to SP600125^16,17^. These results in combination with our findings suggest that Reversine and SP600125 share the common target kinase JNK.

It is becoming increasingly clear that JNK1 is essential for cell migration. In the present study, we found that treatment with SP600125 of intravenously injected tumor cells reduces liver and lung tumor metastases. This effect is in line with previous findings that showing the same effect upon treatment of animals with Reversine^48^. Several studies investigated the effect of JNK1 inhibition by chemicals, small interfering RNA, or dominant-negative mutant, and could confirm the role of JNK-signaling in migration of several different cell types. In these studies, the overexpression of a dominant-negative mutant of JNK1 reduced the migration of rat urinary bladder NBT-II cells^49^, cortical neurons^50^, human umbilical vein endothelial cells^51^, and bovine aortic endothelial cells^52^. Moreover, JNK1 siRNA transfection delays the migration of gastric cancer cells^53^, mouse hepatocellular carcinoma cells^54^, and human melanoma cells^55^. Furthermore, JNK inhibitors were vastly employed to inhibit migration in several cell lines including human dermal fibroblasts^56^, smooth muscle cells^57^, murine cortical neuronal cells^50^, schwann cells^58^, murine epidermal keratinocytes, murine dermal fibroblasts^59^, thyroid cancer cells^60^ uterine luminal epithelial cells^61^ and human lung adenocarcinoma cells^62^.

The JNK signaling is implicated in cell scattering during epithelial-to-mesenchymal transition (EMT) in pulmonary epithelial cells^63^. Moreover, JNK1 is an important regulator of cell-cell adhesion. Indeed, the epithelial tight junctions (TJ) and adherent junctions (AJ) reassembly is accelerated by JNK1 but not by JNK2 inhibition, leading to changes in epithelial morphology and migration delay^64^. In addition, cytoskeletal proteins are important JNK substrates, including proteins associated with the microtubules (DCX, MAP1B, MAP2, Tau and WDR62), proteins associated with the actin cytoskeleton (MARCKSL1 and SMTL2), and focal adhesion proteins (paxillin and β-catenin)^65^. Moreover, recent studies have connected additional JNK target proteins to the cytoskeleton control, including actin filament assembly proteins (Formin1, FHOD3), microtubule-associated proteins (CCSER1), and molecular motors (Myosin-9B). However, these important processes remain largely limited and further studies are needed to explore the mechanism^65^.

In cancer, JNK is widely documented as implicated in several tumors including melanoma, head and neck, breast, ovarian and gastric cancers, suggesting that JNK may be an attractive target for cancer therapy^66^. However, it has also been proposed that JNK1 and JNK2 have different specific cellular targets in cancer and thus the mechanisms involving individual JNK proteins is generally unknown. In the present study we could provide evidence that JNK1 but not JNK2 promotes migration and metastasis of colon carcinoma cells and that Reversine inhibits migration of the cells by modulating the JNK signaling pathway.

## Materials and Methods

### Cell lines, culture conditions and reagents

Human colon carcinoma RKO cells were grown in McCoy’s 5 A medium supplemented 10% fetal calf serum (FCS), 10 mM HEPES buffer, 100 units/mL penicillin G sodium and 100 μg/mL streptomycin sulfate. IMR90 human fibroblast cells were cultured in Dulbecco’s modified Eagle’s medium (DMEM) supplemented with 10% fetal calf serum (FCS) and 100 units/mL penicillin G sodium. NCM460 cells were maintained in INCELL’s enriched M3:10 medium (M310A; which is M3 Base medium plus supplements with 10% FBS and contains antibiotics). Cell lines were routinely maintained at 37 °C under 5% CO_2_. Reversine (BioNordika, Stockholm, Sweden), SP600125 (Sigma-Aldrich), Nocodazole (Sigma-Aldrich) and JNK inhibitor IX (Santa Cruz Biotechnology, Dallas, USA) were stocked as 10 mM solution in DMSO.

### Migration and Invasion assays

For wound-healing assay, scratches were performed on confluent cell monolayers with sterile 200 μl tips and monitored every 12 h by microscopy. Migration distances were expressed as percentages over control values. Indeed, as the scratches are not equal at time 0, we normalized the values by dividing every value by the time 0 value in every condition (Remaining free area/free area time 0).

The two dimensional Oris^TM^ cell migration assays were performed according to the manufacturer’s instructions (Platypus Technologies, Madison, USA). Briefly, cells were seeded (4 × 10^4^ cells per well) into 96-well plates with “silicone stopper” and grown overnight. Then stoppers were removed, and cells were incubated for an GraphPad Prism using ANOVA with Tukey’s test as post-hoc and T-test (for the animal experiments).

## Electronic supplementary material

Supplementary Figures and Information

Original Western blot
