# Supplementary material for: Reversine inhibits Colon Carcinoma Cell Migration by Targeting JNK1
Source: Sci Rep. 2018 Aug 7;8:11821. doi: 10.1038/s41598-018-30251-w (PMC6081478; doi:10.1038/s41598-018-30251-w)
Supplement: Supplementary file 1 — Supplementary Figures and Information [file 41598_2018_30251_MOESM1_ESM.pdf]

Supplementary Figures and Information

Reversine inhibits Colon Carcinoma Cell Migration by Targeting JNK1

Mohamed Jemaà[1,2], Yasmin Abassi[1], Chamseddine Kifagi[3], Myriam Fezai[2], Renée Daams[1], Florian Lang[2,4*] and Ramin Massoumi[1*]

[1]Department of Laboratory Medicine, Translational Cancer Research, Lund University, Lund 22381, Sweden.

[2]Department of Physiology I, Tübingen University, Tübingen, Germany; Gmelinstr. 5, D-72076 Tübingen, Germany.

[3]Division of Immunology and Vaccinology, Technical University of Denmark, Copenhagen, Denmark.

[4]Department of Molecular Medicine II, Medical Faculty, Heinrich Heine University, Duesseldorf, Germany.

Figure S1. **Screening and identification of Reversine and SP600125 as anti-migration inhibitors in sarcoma cells.**

Malignant fibrous histiocytomas MFH152 cells were cultured for 24 h using the Oris™ cell migration assay in the absence or presence of 27 compounds from an in-house and known bioactive molecules targeting mitosis and cell cycle actors at 3 increasing concentrations, 0.1, 1 and 10 µM. Cells were fixed with 4% paraformaldehyde and co-stained with phalloidin and DAPI for the shape and nuclei identification. To identify an efficient anti-migratory agent, we depicted a gate sized by 50% migration and 30% death as compared with non-treated cells, then we considered all the molecules found in this gate as anti-migratory one (**A, B and C**). The screen analysis is based essentially on cell count. First we combined the filled area (phalloidin staining) as a parameter to evaluate the migration with the total number of nucleus (DAPI staining) as a parameter to evaluate the cytotoxicity (**A**). Second, we calculated the number of nucleus inside the migration zone and fixed it as the migration parameter and the total number of nucleus as a parameter to evaluate the cytotoxicity (**B**). Panel (**C**) displays quantitative data of the combined analysis. Grey hits represent the data of covered area analysis while blue hits represent the nuclei count analysis. Panel (**D**) exhibits the list of hit compounds that inhibit cell migration without inducing cell death in both analysis methods. Please note that only two compounds, Reversine and SP600125 (labelled in red), inhibited efficiently cells migration using the two methods of analysis. We also found that Cytochalasin B displays such effect. Cytochalasin is a well-established inhibitor of migration, and *de facto* constitute a good control.

Figure S2. Reversine and SP600125 do not stop normal cells proliferation.
Normal human colon mucosal epithelial cell line NCM460 was seeded in 96 well plates and cultured for 24 h with or without treatment. Cell proliferation was assessed using crystal violet assay. Quantitative data are shown. Grey bar depicts the optic density at 0 h while dark grey bar represents the optic density at 24 h, respectively. Data are reported in SEM; n = 3. n.s (non significant) indicates statistical difference from the absence of Reversine or SP600125 treatment (ANOVA).

Figure S3. Gene ontology analysis associated to Reversine and SP600125 target proteins.

Gene ontology (GO) network of Reversine and SP600125 target proteins was performed using the Cytoscape associated plugin 'ClueGO'. GO network-based enrichment was done following the "representative pathways" parameters (GO levels: 3-8, Minimum 3 genes/term and mapped genes represent at least 4% from the total associated genes). Functionally grouped network with 'GO-nodes' linked was based on their kappa score level (≥0.4), where only the label of the most significant term per group is shown. The node size represents the term enrichment significance overlap.

Figure S4. Knockdown of JNK1 and JNK2 efficiency.
A-B. Efficacy of siRNA-mediated target downregulation of JNK1/2 and JNK2 in colon carcinoma RKO cells. **A.** Total JNK antibody is used. **B.** JNK2 specific antibody is used. Glyceraldehyde-3-phosphate dehydrogenase (GAPDH) levels were monitored to ensure equal loading of lanes. siUNR, control siRNA.

Figure S5. Inactivation of JNK by SP600125 in vivo.
A-B. Inactivation of JNK by SP600125 was evaluated *in vivo* by Western blot. (**A**) shows total and phospho JNK1/2 in liver and lung tissue extracted from mice treated with vehicle or SP60125 while (**B**) depict quantitative data of phospho JNK protein/GAPDH ratio.

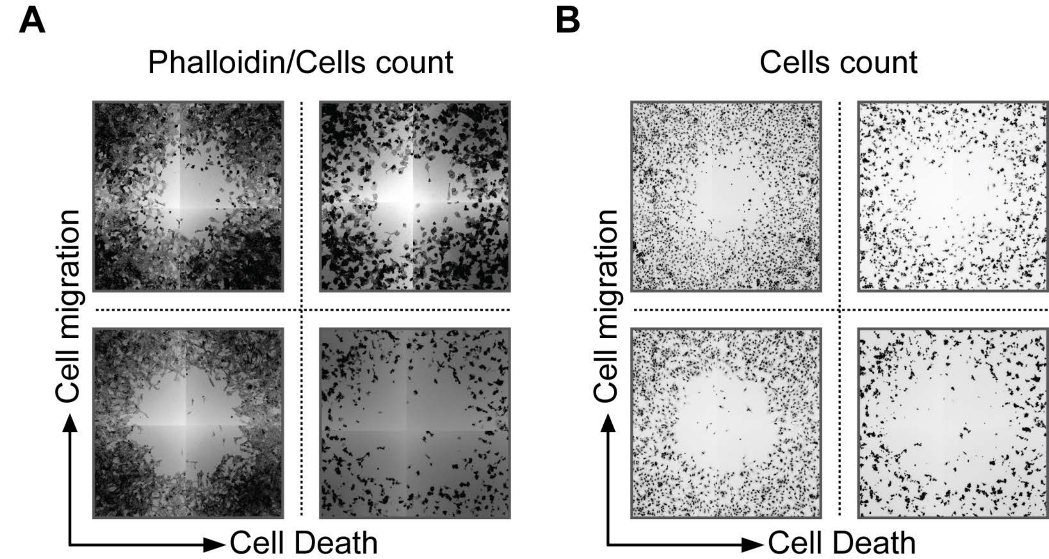

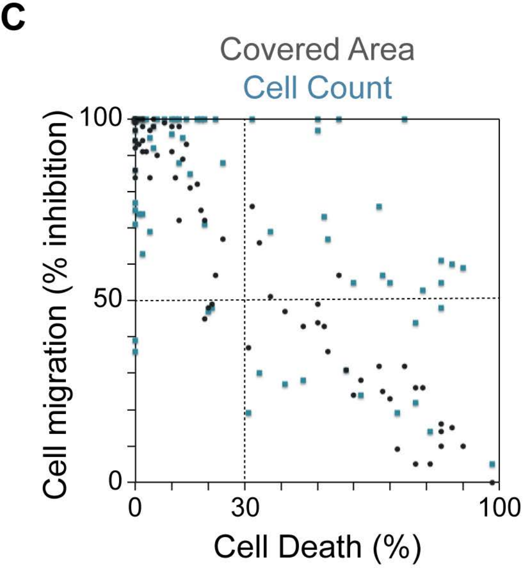

D

Covered Area
Reversine
SP600125
ITX3 (Trio N-Terminal RhoGEF inhibitor)
**Cell Count**
Reversine
SP600125
Cytochalasin B
CDK1 inhibitor

SUPPLEMENTARY FIGURE 1

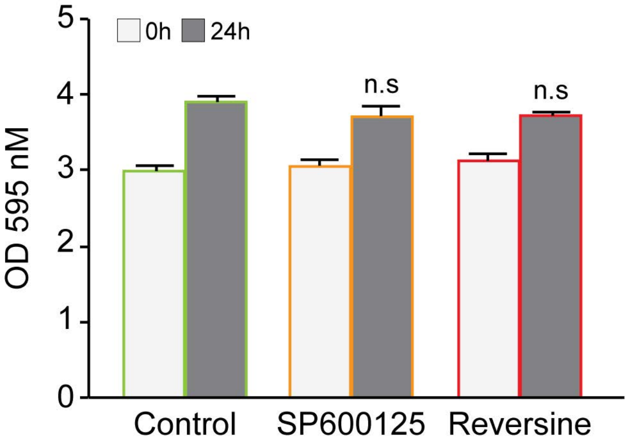

SUPPLEMENTARY FIGURE 2

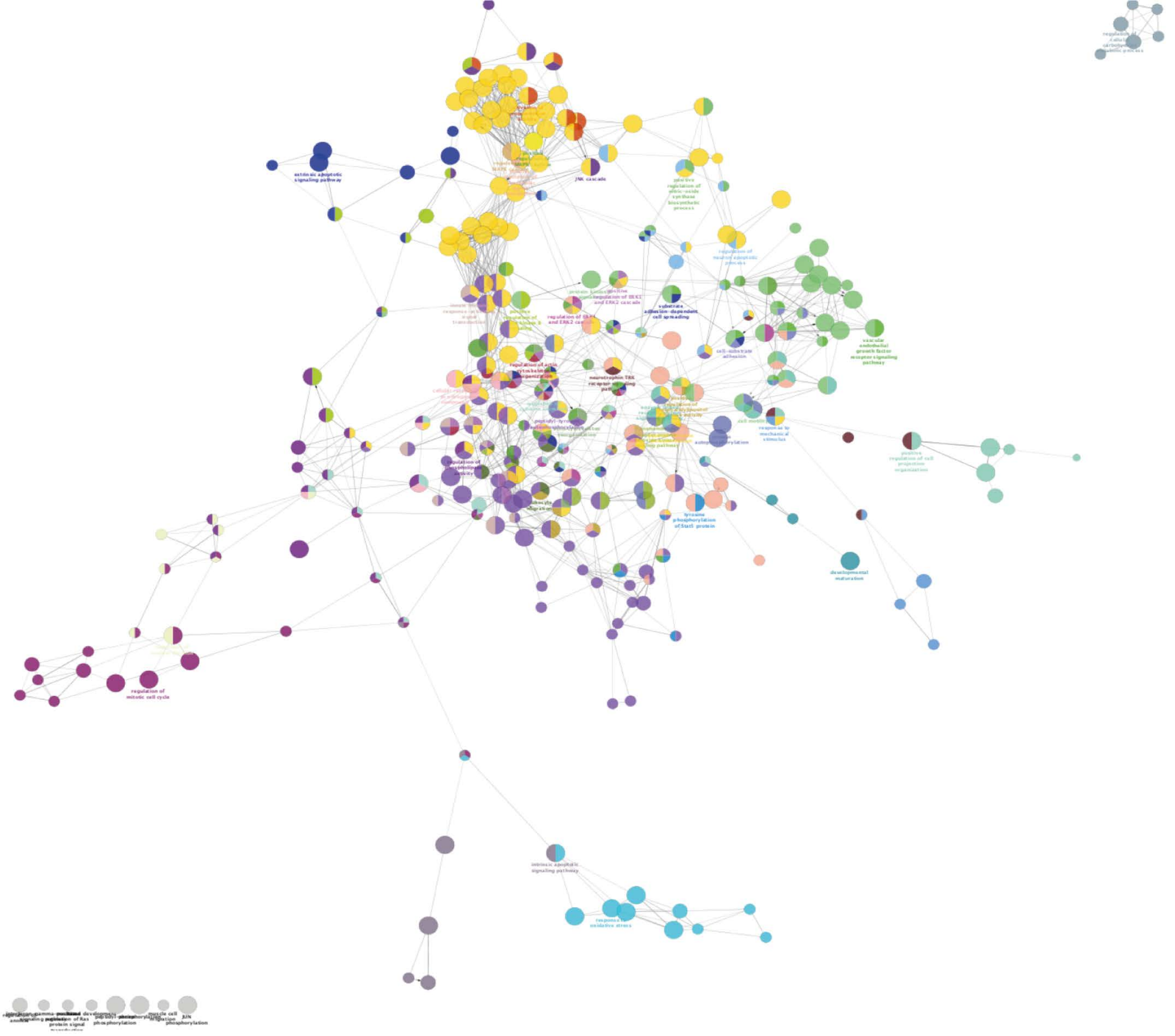

SUPPLEMENTARY FIGURE 3

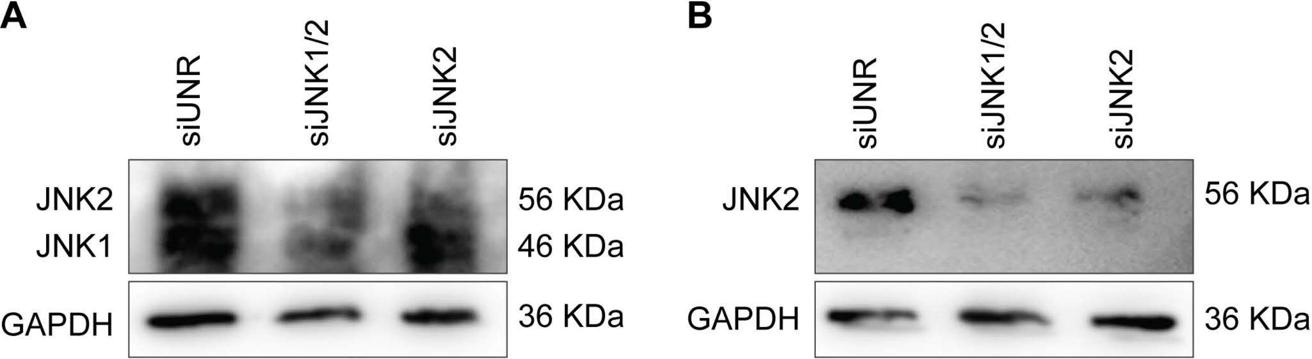

SUPPLEMENTARY FIGURE 4

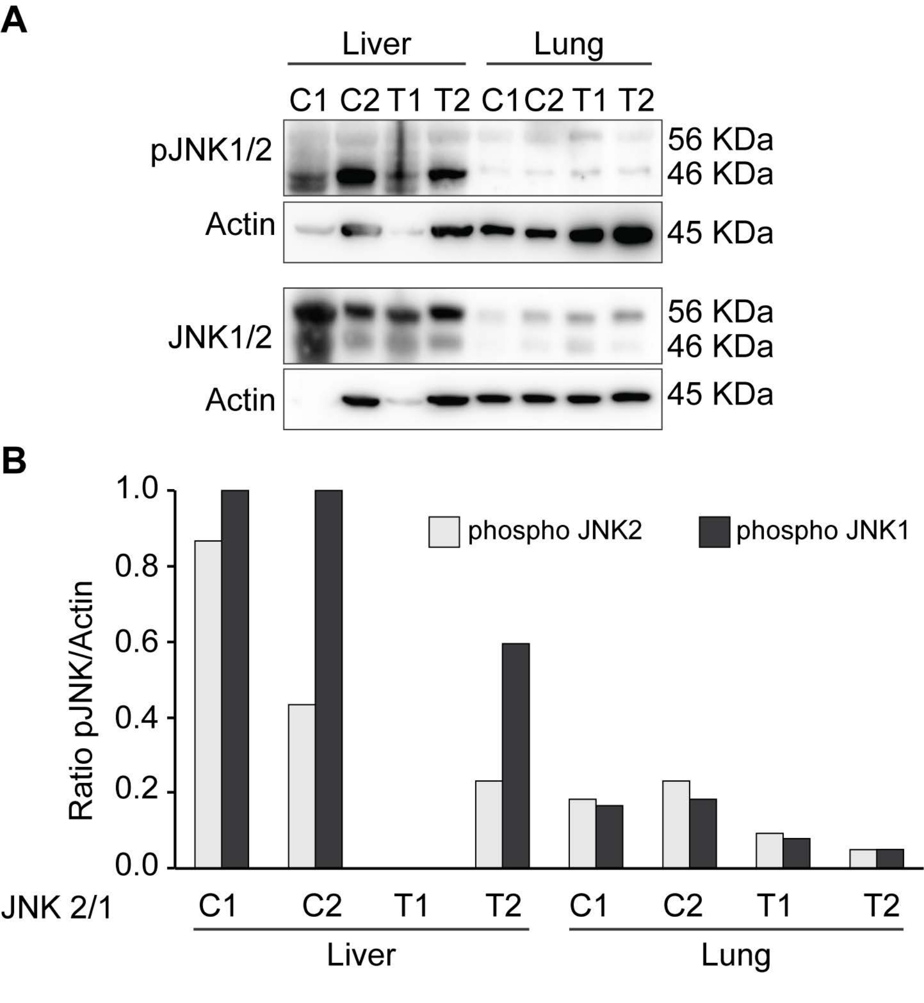

SUPPLEMENTARY FIGURE 5
