# Supplementary material for: Reversine inhibits Colon Carcinoma Cell Migration by Targeting JNK1
Source: Sci Rep. 2018 Aug 7;8:11821. doi: 10.1038/s41598-018-30251-w (PMC6081478; doi:10.1038/s41598-018-30251-w)

Supplementary Information

Reversine inhibits Colon Carcinoma Cell Migration by Targeting JNK1

Mohamed Jemaà[1,2], Yasmin Abassi[1], Chamseddine Kifagi[3], Myriam Fezai[2], Renée Daams[1], Florian Lang[2,4*] and Ramin Massoumi[1*]

[1]Department of Laboratory Medicine, Translational Cancer Research, Lund University, Lund 22381, Sweden.

[2]Department of Physiology I, Tübingen University, Tübingen, Germany; Gmelinstr. 5, D-72076 Tübingen, Germany.

[3]Division of Immunology and Vaccinology, Technical University of Denmark, Copenhagen, Denmark.

[4]Department of Molecular Medicine II, Medical Faculty, Heinrich Heine University, Duesseldorf, Germany.

Original Western Blot for figure 4

Figure 4D

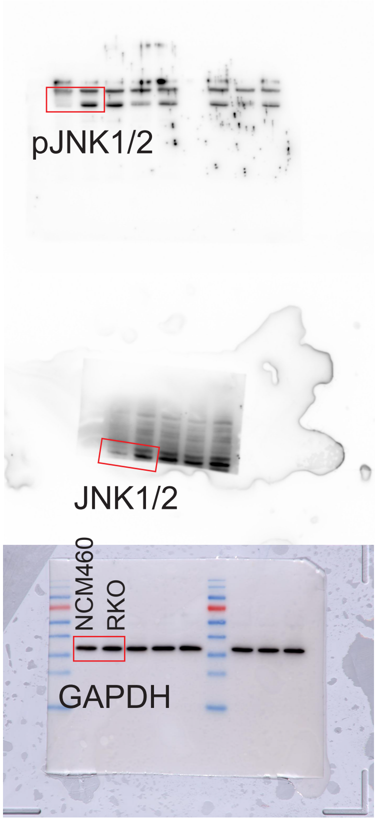

Figure 4E

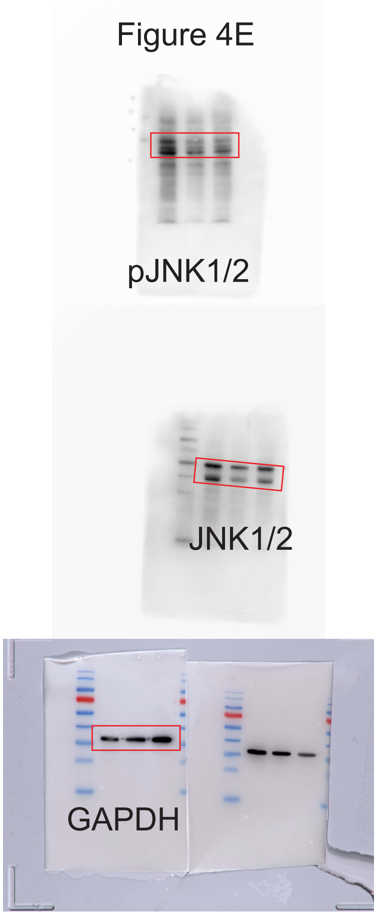

Figure 4F

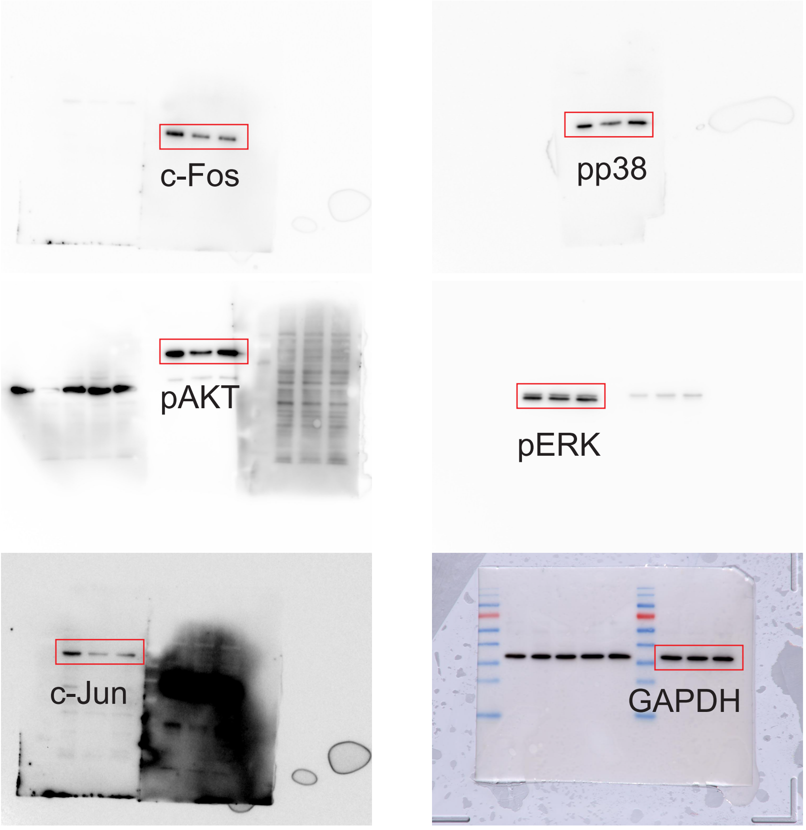

Original Western Blot for figure S4

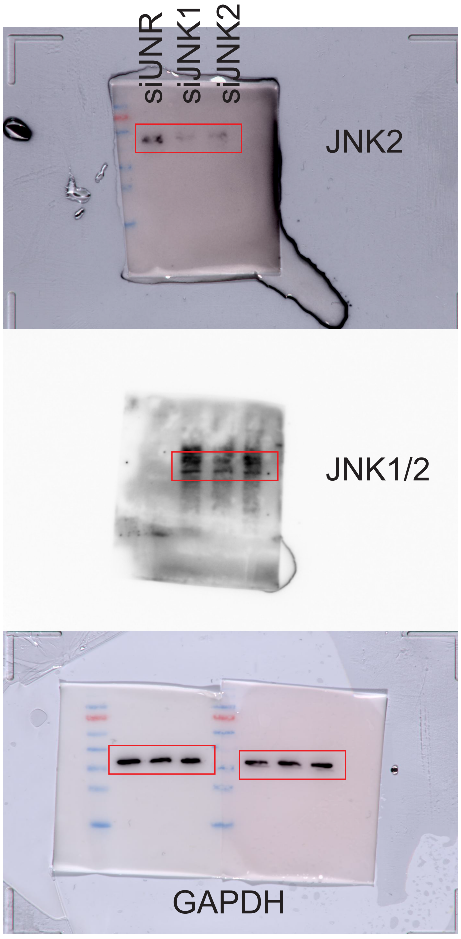

Original Western Blot for figure S5

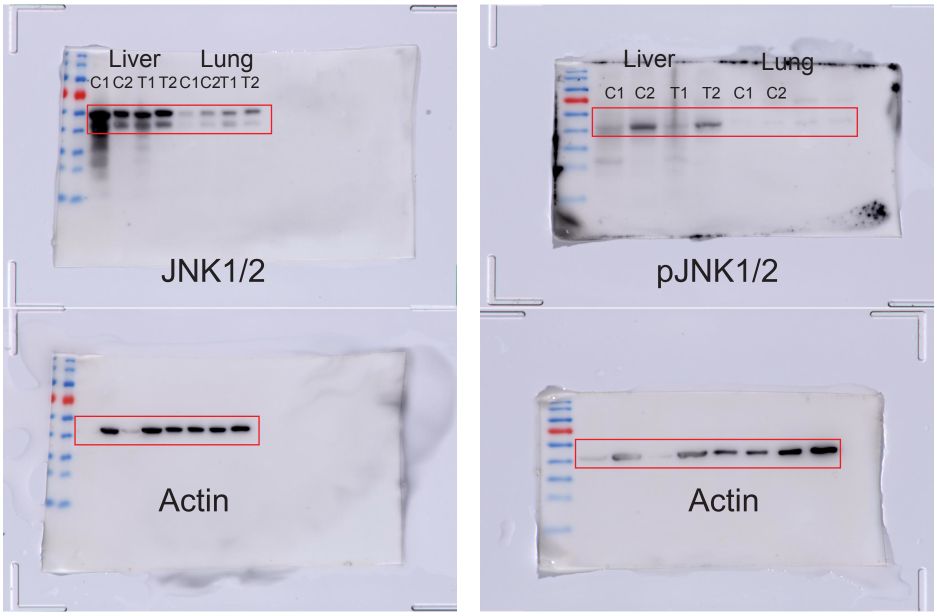

Supplement: Supplementary file 2 — Original Western blot [file 41598_2018_30251_MOESM2_ESM.pdf]
